# Impact of Pharmacist-Led Clinics on Health Outcomes of Patients With Diabetes at a Ministry of Health Diabetes & Endocrinology Center, Saudi Arabia: A Retrospective Study

**DOI:** 10.7759/cureus.25923

**Published:** 2022-06-14

**Authors:** Saleh F Alqifari, Bader AlMharwal, Rahaf Aldawish, Salman A Almokhlef

**Affiliations:** 1 Faculty of Pharmacy, Department of Pharmacy Practice, University of Tabuk, Tabuk, SAU; 2 Diabetes and Endocrinology Center, King Fahad Specialist Hospital, Buraydah, SAU; 3 Department of Clinical Sciences, Sulaiman Alrajhi University, Al-Qassim, SAU

**Keywords:** ambulatory care, disease management, diabetes mellitus, endocrinology, clinical pharmacy

## Abstract

Objectives: Saudi Arabia is one of the most diabetes-prone countries in the world. The physician‐centered model of care constitutes the standard of care around the country. The study aimed to evaluate the impact of clinical pharmacist care on diabetes management in comparison to standard physician-based care.

Materials and methods: a retrospective chart review was conducted of patients with type 2 diabetes mellitus (T2DM) seen by the clinical pharmacist at the Diabetes & Endocrinology Center, King Fahad Specialist Hospital located in Buraydah, Saudi Arabia between September 2019 to June 2020.

Results: Thirty-two diabetic patients were included. The mean age is 55.75±10.72 years with 65.6% of patients being females. Hemoglobin A1c (HbA1c) was significantly decreased within nine months compared to baseline (9.33±1.80 vs.10.30±1.66), *p*=0.017.

Conclusions: The multidisciplinary collaborative care involving pharmacists achieved superior diabetes outcomes for patients with diabetes. Involving pharmacists resulted in a significant HbA1c reduction within nine months. Moreover, pharmacists’ care helped optimize medication therapy and decreased the frequency of hypoglycemia.

## Introduction

Saudi Arabia has experienced enormous economic and lifestyle changes over the past few decades. Along with these changes, there was a rapid increase in the prevalence of diabetes mellitus which is currently a major public health issue in the country [1].

In 2017, Saudi Arabia was ranked second among Middle Eastern countries and seventh in the world for the prevalence of diabetes mellitus by the World Health Organization (WHO). Also in 2017, about seven million individuals were diagnosed with diabetes and approximately three million are considered to have pre-diabetes [2]. An epidemiological community-based national survey conducted among Saudi nationals 30-70 years of age over five years and including 16,917 participants revealed that 4,004 individuals (23.7%) of participants had diabetes [3].

Diabetes complications are another concerning issue in Saudi Arabia. Even though people with diabetes in Saudi Arabia have free access to both good quality healthcare services and medications, the prevalence of microvascular (i.e. retinopathy, neuropathy, and nephropathy) and macrovascular complications (i.e. coronary artery disease (CAD), stroke, and peripheral vascular disease (PVD)) were high [1]. Diabetes complications reduce the quality of life and life expectancy of the affected people and create an economic, emotional, and social disease burden. Several studies from Saudi Arabia investigated traditional risk factors for macro- and microvascular diabetes complications. Most of these studies found that gender, older age, longer duration of diabetes, use of insulin, and poor glycemic control were associated factors [1].

In the U.S., Ourth et al. conducted a cohort study to evaluate the safety and effectiveness of management of hyperglycemia offered by clinical pharmacists compared to that offered through usual physician-centered care in out-patient settings. They included 12,327 patients with hemoglobin A1c (HbA1c) of >9% managed by clinical pharmacists and an equal number managed by primary care physicians. Results revealed that the number of visits to reach an HbA1c level of <8% was lower in the physician-centered group compared to the pharmacist-centered group (1.82±1.27 vs. 2.46±1.58), p <0.001 [4]. Peterson et al. carried out a multicenter cohort study to assess the effect of physician-pharmacist visits on clinical outcomes of adult patients aged 18 years or above with uncontrolled diabetes (HbA1c ≥8%). Results showed that patients managed in the physician-pharmacist visits model had a significant decrease in mean change in HbA1c from baseline to follow-up (p <0.001) [5]. In Slovenia, Schultz et al. carried out a retrospective cohort study to assess the effect of a pharmacist-managed diabetes clinic on clinical outcomes compared to usual care received from primary care providers (PCPs). Results revealed that the absolute change in HbA1c was +1.53% in the phase of usual care versus -1.63% in the intervention phase. Additionally, diabetes-related hospitalizations were reduced from 10 to six and emergency room (ER) visits also decreased from 27 to eight [6].

In Saudi Arabia, diabetes is quickly reaching disturbing proportions and becoming a significant cause of morbidity and mortality. As the prevalence of diabetes increases in Saudi Arabia, the physician-centered approach is making every effort to provide holistic care. However, diabetes can be managed effectively and efficiently with a multidisciplinary collaborative care strategy. Alsuwayni et al. implemented a prospective cohort study to assess a pharmacist-led diabetes clinic’s impact on patients` outcomes (HbA1c level, routine screenings, medication compliance as well as biomarkers of other comorbidities). The baseline results were compared to the three months follow-up results for 35 patients. At follow-up, HbA1c had significantly decreased by 1.2% (p <0.001), and nine patients achieved their HbA1c goal of <7% [7].

Clinical pharmacist intervention in the management of chronic diseases has been shown to improve clinical outcomes. This model of care has been ranked among the most successful multidisciplinary collaborative care models affecting the control of diabetes as well as other chronic diseases. We aim to evaluate the application of this model of care on patients with diabetes in Saudi Arabia.

## Materials and methods

Design and study population 

A retrospective cohort study was conducted between September 2019 to June 2020 via chart review of all adult patients with uncontrolled type 2 diabetes whom the clinical pharmacist saw for at least one visit within the nine-month study span at Al-Qassim Diabetes & Endocrinology Center, Buraydah, Kingdom of Saudi Arabia. The Diabetes & Endocrinology Center sees more than 10,000 patients annually from all around the Alqassim region. The patient population at the diabetes center includes adults and children with type 1 and type 2 diabetes. Patients were referred to the pharmacist-led clinic if they have been formally diagnosed and did not meet their established therapeutic goal set by the physician for more than nine months. A collaborative practice agreement (CPA) was signed between the pharmacist and providers at the clinic to refer patients to the service. The CPA allowed modifications of the therapy plan after a shared decision between the patient and the pharmacist. The study population consisted of uncontrolled T2DM patients aged 18 years or older. A patient is considered uncontrolled if they have an HbA1c value above their established goal at the initial clinic visit. The exclusion criteria were patients with type 1 diabetes mellites or controlled T2DM. The study protocol was granted IRB approval by the Regional Research Ethics Committee - Qassim Province (approval 1443-176248). Patient information was obtained from the electronic medical record CareWare® and de-identified data was captured using the Research Electronic Data Capture (REDCap®).

Data analysis

Demographic data were presented as mean ± standard deviation (SD) for continuous variables, number, and percentage for categorical variables. Statistical significance for continuous variables was analyzed using a paired t-test and McNemar test was used to analyze categorical variables. An alpha-error of 0.05 or less was considered statistically significant. Data entry and analysis were performed utilizing SPSS® version 26 software (IBM Corp., Armonk, NY, USA).

## Results

The study included 32 patients. The mean age was 55.75±10.72 years. Females represented almost two-thirds of participants (65.6%). Baseline HbA1c% was 10.61±1.89% and the mean duration of diabetes was 13.45±7.49 years. At baseline, 62.5% of patients were on insulin therapy, 78.1% on metformin, 28.1 on a sulfonylurea, 53.1% on a dipeptidyl peptidase-4 (DPP4) inhibitor, 6.25% use a glucagon-like peptide-1 receptor (GLP-1) agonist and the same percentage take a sodium-glucose co-transporter-2 (SGLT2) inhibitor. About 46.5% were taking two antidiabetic drugs whereas 32.1% were taking three drugs at baseline. The mean of self-reported hypoglycemia frequency was 1.5 episodes per week at baseline. The mean total daily insulin dose was 57.4±29.7 units (Table 1). 

**Table 1:** Baseline characteristics of participants (n=32)

Characteristics	Value
Age, years	55.75 ±10.72
Gender, n (%)	
Female	21 (65.6%)
Body Mass Index (BMI), kg/m^2^	30.1 ±5.75
Hemoglobin A1c (HbA1c), %	10.61% ±1.89
Duration of DM, years	13.45 ±7.49
Antihyperglycemic drug therapy, n (%)	
Insulin	20 (62.5%)
Biguanide	25 (78.1%)
Sulfonylurea	9 (28.1%)
Dipeptidyl peptidase-4 (DPP-4) inhibitor	17 (53.1%)
Glucagon-like peptide-1 (GLP-1) agonist	2 (6.25%)
Sodium-glucose co-transporter-2 (SGLT-2) inhibitor	2 (6.25%)
Hypoglycemia frequency, episode/week	1.5
Insulin total daily dose, units	57.4±29.7

Within nine months, the HbA1c percentage was significantly decreased compared to the baseline, 9.33±1.80 vs. 10.30±1.66, p=0.017. Moreover, the composition of the number of oral antidiabetic agents significantly changed at nine months where no patients were left on one oral antidiabetic agent, 18 patients were on two agents, 11 patients on three agents, and only three patients on four agents (p=0.033). Insulin utilization insignificantly decreased from 57.4±29 to 55±31.0 units in total daily dose (p=0.462). A slight nonsignificant decrease in BMI was noted at nine months from 31.23±4.68 to 30.1±5.75 (p=0.258) (Table 2).

**Table 2:** Comparison between baseline (physician-centered) and within nine months (Clinical pharmacist-centered) patient parameters ⱡ Paired t-test             *McNemar test

Variable	Baseline	Within 9 months	p-value
Hemoglobin A1c (HbA1c), %	10.30±1.66	9.33±1.80	0.017^ⱡ^
Body Mass Index (BMI), kg/m2, mean (SD)	31.23 ±4.68	30.1 ±5.75	0.258^ⱡ^
Insulin total daily dose, units	57.4 ±29.7	55 ±31.0	0.462^ⱡ^
Number of oral antidiabetic agents, No. (%)			
One	7 (21.9)	0 (0)	0.033*
Two	15 (46.9)	18 (56.3)	
Three	10 (31.3)	11 (34.4)	
Four	0 (0)	3 (9.4)	

Overall, utilization of sulfonylureas in nine patients (28.1%) decreased to five (15.6%) at nine months. Moreover, all patients were optimized on metformin within nine months. Utilization of DPP-4 inhibitors slightly decreased from 17 (53.1%) to 16 (50%) while utilization of GLP-1 agonists and SGLT-2 inhibitors increased from two (6.3%) to eight (25%) and two (6.3%) to six (18.8%) respectively. Average hypoglycemia frequency decreased from 1.5 episodes per week to 0.5 at nine months (Table 3).

**Table 3:** Comparison between baseline (physician-centered) and within nine months (Clinical pharmacist-centered) patient medications and hypoglycemia frequency

Variable	Baseline	Within 9 months
Antihyperglycemic drug therapy, n (%)		
Insulin	20 (62.5%)	22 (68.8%)
Biguanide	25 (78.1%)	32 (100%)
Sulfonylurea	9 (28.1%)	5 (15.6%)
Dipeptidyl peptidase-4 (DPP-4) inhibitor	17 (53.1%)	16 (50%)
Glucagon-like peptide-1 (GLP-1) agonist	2 (6.3%)	8 (25%)
Sodium-glucose co-transporter-2 (SGLT-2) inhibitor	2 (6.3%)	6 (18.8%)
Hypoglycemia frequency, episode/week	1.5	0.5

## Discussion

Clinical pharmacists as healthcare providers have demonstrated a positive effect on the overall healthcare quality [8-11]. Throughout Saudi Arabia, there is an increase in the prevalence of diabetes and its complications with the expected increase in the upcoming years. Therefore, clinical pharmacists can be a noble asset in the management of diabetes and its complications in Saudi Arabia. The present study aimed to evaluate the impact of pharmacists on diabetes care compared to the standard model with physician-based care in Saudi Arabia. The study has shown significant improvements in diabetes control as evidenced by lowering HbA1c, lowering of frequency of hypoglycemia, and transitioning patients to newer antihyperglycemic agents after nine months of care by clinical pharmacists compared to physician care. Our observations are consistent with what has been observed in recent similar studies carried out in Saudi Arabia, Jordan, Slovenia, and United States as well as recent systematic reviews and meta-analyses [4,7,10,12-14]. 

The observed improvement in HbA1c values over nine months period in the current study confirms the value of the role that could be played by clinical pharmacists in the care of diabetic patients in collaboration with healthcare providers to offer the best care for those patients. The integration between practices of both clinical pharmacists and physicians has been proven to produce a positive impact on patient care in other settings as well [13].

Fink et al. revealed numerous differences between the practices of clinical pharmacists and usual care in managing patients with uncontrolled T2DM. One of which is that clinical pharmacists were more likely to prescribe a non-insulin medication and start newer antihyperglycemic drugs than usual care. Furthermore, clinical pharmacists are less likely to have no medication changes at all compared to usual care [14]. The difference in prescribing patterns between clinical pharmacists and usual care could be explained by the nature of the clinical visits and the type of the provider of care. Moreover, patients cared for by a physician may receive care from different providers at each visit compared to clinical pharmacist clinics. 

Clinical inertia is a term used to describe “the lack of treatment intensification despite not meeting glycemic goals” in the management of diabetes [15,16]. It has been documented that clinical pharmacists were more likely than usual care providers to intensify treatment [15]. Clinical pharmacists are adequately trained in various aspects of drug management, including clinical use and adverse effects [17,18]. It has been observed that intensification of therapy might reduce intervals of uncontrolled hyperglycemia and decrease the risk of diabetes-linked complications [8]. Furthermore, it has been reported that clinical pharmacists were more likely than usual care providers to follow evidence-based practices and reduce drug-related problems [10,14,19].

Although pharmacists’ involvement in clinical settings can produce better outcomes, it is imperative to emphasize that challenges exist in applying this model of care. Such challenges include poor documentation of patient history and medical information, preconceived notions of patients regarding specific drugs such as insulin, and drug shortages, especially for newer antihyperglycemic agents. This hinders the pharmacist's efforts to start and maintain patients on newer therapies such as SGLT-2 inhibitors, GLP-1 receptor agonists and insulin. 

Limitations of the study

Although the findings of this study are consistent with findings of other similar studies, it is a single-center study which can impact the external validity of its findings. Furthermore, the small sample size is typical of pharmacists' clinics due to the nature and scope of the practice as pharmacists provide care for selected patients with diabetes compared to physicians. Care by other healthcare providers, such as dietitians and social workers within the diabetes center or externally may influence the outcome of care in both groups. Adherence to medications prescribed by the diabetes center or other external providers could not be included in the present study due to information unavailability as patients may see other external providers during the study span of nine months. Moreover, pharmacy and refill records of prescriptions are not available for inclusion in the present study. 

## Conclusions

The multidisciplinary collaborative care involving pharmacists achieved superior diabetes outcomes for patients with type 2 diabetes. Involving pharmacists resulted in a significant HbA1c reduction within nine months. Moreover, pharmacists’ care helped optimize therapeutic outcomes and decreased the frequency of hypoglycemia.

## References

[1] Alramadan MJ, Magliano DJ, Alhamrani HA (2019). Lifestyle factors and macro- and micro-vascular complications among people with type 2 diabetes in Saudi Arabia. Diabetes Metab Syndr.

[2] Al Dawish MA, Robert AA, Braham R, Al Hayek AA, Al Saeed A, Ahmed RA, Al Sabaan FS (2016). Diabetes mellitus in Saudi Arabia: a review of the recent literature. Curr Diabetes Rev.

[3] Naeem Z (2015). Burden of diabetes mellitus in Saudi Arabia. Int J Health Sci (Qassim).

[4] Ourth HL, Hur K, Morreale AP, Cunningham F, Thakkar B, Aspinall S (2019). Comparison of clinical pharmacy specialists and usual care in outpatient management of hyperglycemia in Veterans Affairs medical centers. Am J Health Syst Pharm.

[5] Peterson J, Hinds A, Garza A (2020). Impact of physician-pharmacist covisits at a primary care clinic in patients with uncontrolled diabetes. J Pharm Pract.

[6] Schultz JL, Horner KE, McDanel DL (2018). Comparing clinical outcomes of a pharmacist-managed diabetes clinic to usual physician-based care. J Pharm Pract.

[7] Alsuwayni B, Alhossan A (2020). Impact of clinical pharmacist-led diabetes management clinic on health outcomes at an academic hospital in Riyadh, Saudi Arabia: a prospective cohort study. Saudi Pharm J.

[8] McBane SE, Dopp AL, Abe A (2015). Collaborative drug therapy management and comprehensive medication management-2015. Pharmacotherapy.

[9] Alhossan A, Kennedy A, Leal S (2016). Outcomes of annual wellness visits provided by pharmacists in an accountable care organization associated with a federally qualified health center. Am J Health Syst Pharm.

[10] Nasution A, Dalimunthe A, Khairunnisa K (2019). Pharmacists intervention reduced drug-related problems in the treatment of patients with type 2 diabetes mellitus. Open Access Maced J Med Sci.

[11] Khdour MR, Agus AM, Kidney JC, Smyth BM, McElnay JC, Crealey GE (2011). Cost-utility analysis of a pharmacy-led self-management programme for patients with COPD. Int J Clin Pharm.

[12] Carter BL, Ardery G, Dawson JD (2009). Physician and pharmacist collaboration to improve blood pressure control. Arch Intern Med.

[13] van Eikenhorst L, Taxis K, van Dijk L, de Gier H (2017). Pharmacist-led self-management interventions to improve diabetes outcomes. A systematic literature review and meta-analysis. Front Pharmacol.

[14] Fink RM, Mooney EV, Saseen JJ, Billups SJ (2019). A comparison of clinical pharmacist management of type 2 diabetes versus usual care in a federally qualified health center. Pharm Pract (Granada).

[15] Okemah J, Peng J, Quiñones M (2018). Addressing clinical inertia in type 2 diabetes mellitus: a review. Adv Ther.

[16] Reach G, Pechtner V, Gentilella R, Corcos A, Ceriello A (2017). Clinical inertia and its impact on treatment intensification in people with type 2 diabetes mellitus. Diabetes Metab.

[17] Saseen JJ, Ripley TL, Bondi D (2017). ACCP clinical pharmacist competencies. Pharmacotherapy.

[18] Bailey CJ (2016). Under-treatment of type 2 diabetes: causes and outcomes of clinical inertia. Int J Clin Pract.

[19] Jarab AS, Alqudah SG, Mukattash TL, Shattat G, Al-Qirim T (2012). Randomized controlled trial of clinical pharmacy management of patients with type 2 diabetes in an outpatient diabetes clinic in Jordan. J Manag Care Pharm.

